# Research on Strong Clutter Suppression for Gaofen-3 Dual-Channel SAR/GMTI

**DOI:** 10.3390/s18040978

**Published:** 2018-03-26

**Authors:** Mingjie Zheng, He Yan, Lei Zhang, Weidong Yu, Yunkai Deng, Robert Wang

**Affiliations:** 1 Space Microwave Remote Sensing System Department, Institute of Electronics, Chinese Academy of Sciences, Beijing 100190, China; 314forever@163.com (L.Z.); ywd@mail.ie.ac.cn (W.Y.); ykdeng@mail.ie.ac.cn (Y.D.); yuwang@mail.ie.ac.cn (R.W.); 2 Institute of Electronic Information Engineering, Nanjing University of Aeronautics and Astronautics, Nanjing 210006, China; yanhe@nuaa.edu.cn

**Keywords:** Gaofen-3, SAR/GMTI, clutter suppression, error correction

## Abstract

In spaceborne synthetic aperture radar (SAR), moving targets are almost buried in ground clutter due to the wide clutter Doppler spectrum and the restricted pulse repetition frequency (PRF), which increases the difficulty of moving target detection. Clutter suppression is one of the key issues in the spaceborne SAR moving target indicator operation. In this paper, we describe the clutter suppression principle and analyze the influence of amplitude and phase error on clutter suppression. In the following, a novel dual-channel SAR clutter suppression algorithm is proposed, which is suitable for the Gaofen-3(GF-3) SAR sensor. The proposed algorithm consists of three technique steps, namely adaptive two-dimensional (2D) channel calibration, refined amplitude error correction and refined phase error correction. After channel error is corrected by these procedures, the clutter component, especially a strong clutter component, can be well suppressed. The validity of the proposed algorithm is verified by GF-3 SAR real data which demonstrates the ground moving-target indication (GMTI) capability of GF-3 SAR sensor.

## 1. Introduction

Synthetic aperture radar (SAR) ground moving-target indication (GMTI) technique is becoming more and more important. There have been more SAR sensors which are capable of detecting moving targets, such as Radarsat-2 and TerraSAR-X [1,2,3]. The Gaofen-3 (GF-3) SAR satellite is the first C-band multi-polarization synthetic aperture radar imaging satellite which was launched on 10 August 2016 in China. The GF-3 SAR has 12 imaging modes. The resolution ranges from 1 m to 500 m, while the corresponding swath ranges from 10 km up to 650 km. In addition to these imaging modes, GF-3 SAR has several test modes, including SAR/GMTI mode, terrain observation by progressive scans (TOPS) mode, high resolution-wide swath (HRWS) mode and extended sliding spot mode [4,5,6,7]. The SAR/GMTI mode is designed based on fine strip I (FSI) with a resolution of 5 m and a swath of 50 km, and is a dual receiving channel mode which has the same working way with the ultra-fine strip (UFS) [8,9].

In spaceborne SAR, the clutter Doppler frequency spectrum is very wide due to the high velocity of the platform. Therefore, most moving targets are buried in ground clutter. For multi-channel SAR sensors, moving targets can be detected after clutter is suppressed [10,11]. The conventional GMTI techniques include the displaced phase center antenna (DPCA) technique [12], extended DPCA (EDPCA) [13], signal subspace projection (SSP) technique [14], along-track interferometry(ATI) technique [15,16], clutter suppression interferometry (CSI) technique [17,18], and the space–time adaptive processing (STAP) technique [19,20]. Usually, moving target detection operation can be carried out in the range-Doppler domain or image domain.

In spaceborne SAR, due to the restriction of the zebra map, pulse repetition frequency (PRF) is usually not allowed to be very high in compromise with the swath width. The higher the PRF, the narrower the swath width. The limited PRF results in the folding of the clutter spectrum, which raises the clutter power, which is bad for moving target detection. Therefore, when the clutter spectrum is aliasing, the greater the channel number, the stronger the clutter suppression ability. However, due to the limitations of weight, power and cost, most spaceborne SAR/GMTI sensors have only two channels—such as TerraSAR-X and Radarsar-2—which causes clutter suppression performance degradation, especially in the presence of strong clutter. Due to variation of a satellite’s velocity and attitude, and the difference of channel transfer functions, there are amplitude and phase errors between channels, which will further aggravate the clutter suppression performance. The strong clutter component cannot be well suppressed due to the channel error, and the residue clutter component after channel cancellation can be detected as moving targets by the constant false alarm rate (CFAR) detector.

In this paper, we describe the clutter suppression principle and set up the clutter suppression model. The influence of amplitude and phase error on clutter suppression is analyzed. A novel algorithm is proposed to suppress strong ground clutter for dual-channel spaceborne SAR/GMTI sensors based on high-accuracy amplitude and phase error correction. First, the adaptive two-dimensional (2D) channel calibration is performed to calibrate the difference between channels. Then the refined amplitude correction is performed cell-by-cell to balance the two channels’ amplitude. At last, refined phase correction based on selected strong clutter is performed to correct channel phase error. Through these procedures, channel error is gradually corrected. Compared with conventional techniques, the proposed algorithm has better performance in suppressing strong clutter, especially in artificial buildings, bridges, high mountains, etc. The proposed algorithm can be applied in the image domain or the two-dimension frequency domain.

The rest of the paper is organized as follows. In Section 2, the clutter suppression principle and model are described and the influence of amplitude and phase error on clutter suppression is analyzed. In Section 3, the algorithm is proposed and analyzed in more detail. In Section 4, the performance of the proposed algorithm is verified by GF-3 SAR experiment data. Finally, Section 5 concludes the paper.

## 2. Clutter Suppression Principle and Model

**Figure 1:**
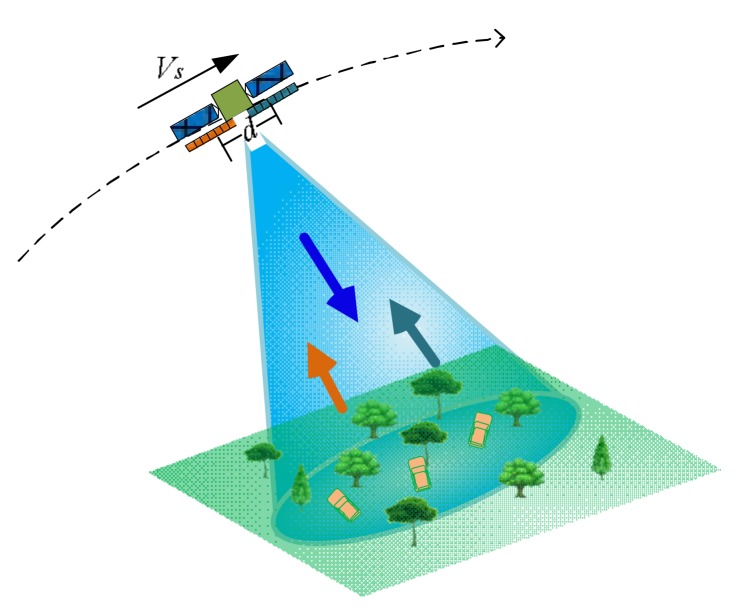
Schematic of GF-3 SAR/GMTI.

For SAR/GMTI mode, the sensor transmits a signal with full aperture and receives an echo with two sub-apertures simultaneously. Each sub-aperture is half of the full aperture and is placed along track. The schematic is briefly shown in Figure 1, in which *V_s_* denotes the satellite velocity.

The received echo of the two channels is composed of moving targets, clutter and noise. After imaging, we obtain the signal model in the image domain as the following equation:

(1) $$Z_{i}=S_{i}+C_{i}+N_{i}\;i=1,2$$

where *Z* denotes the received echo of the two channels, *S* denotes moving targets, *C* denotes clutter, *N* denotes noise, and the index *i* denotes the channel number.

Ideally, clutter is consistent between channels and moving targets are different because of movement. However, due to all kinds of system error, such as radar transceiver equipment error, satellite attitude error, system measurement error, and the aperture displacement, there are amplitude and phase errors between channels, which lead to channel unbalance. Therefore, the relationship model between the two channels is written in the following equation:

(2) $$Z_{2}=(S_{1}\cdot e^{j\varphi }+C_{1}+N_{2})\cdot Ae^{j\theta }$$

where *A* denotes amplitude error between channels, *θ* denotes phase error between channels, and *ϕ* denotes the interferometric phase caused by target movement.

The discrepancy of moving targets between channels is mainly caused by the channel error and target movement, and the discrepancy of clutter is mainly caused by the channel error, and noise is random. The channels are error free when *A* = 1 (0 dB) and *θ* = 0. In practice, the definite amplitude and phase error can be obtained and compensated through the system test, and the indefinite error is mainly caused by various random factors, such as measurement error. The indefinite error causes the clutter cancellation performance degradation.

*φ* is represented by the following formula:

(3) $$\varphi =\frac{4\pi \cdot d\cdot v_{r}}{2\lambda \cdot V_{s}}$$

where *v_r_* is the moving target radial velocity, *V_s_* is the sensor velocity, *d* is the distance between two sub-apertures, and *λ* is the wave length.

Due to the amplitude and phase error between channels, clutter cannot be completely suppressed. According to Equations (1)–(3), the signal-to-clutter-noise ratio (SCNR) is written as follows after clutter suppression:

(4) $$SCNR=\frac{1+A^{2}-2A\cos(\varphi +\theta )}{\frac{1}{SCR_{b}}(1+A^{2}-2A\cos(\theta ))+\frac{1}{SNR_{b}}(1+A^{2})}$$

where *SCR_b_* is signal clutter ratio before clutter suppression, and *SNR_b_* is signal noise ratio before clutter suppression. As can be seen from Equation (4), *SCNR* is affected by the amplitude and phase error between channels, affected by the signal-to-clutter ratio (SCR) and signal-to-noise ratio (SNR) before clutter suppression, and also affected by the interferometric phase caused by target movement.

**Figure 2:**
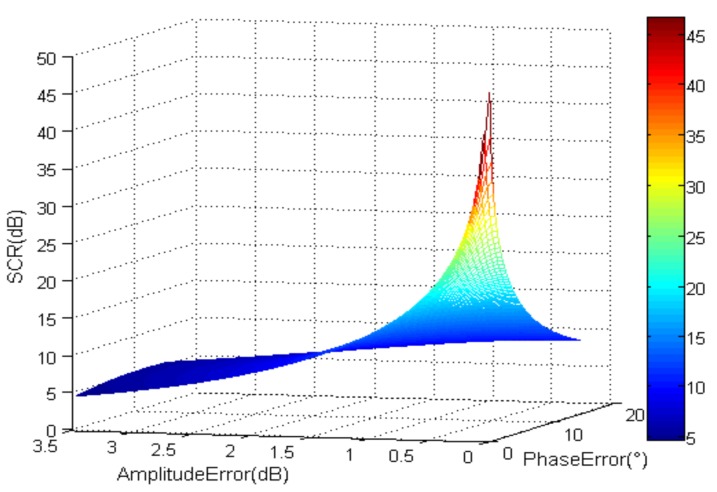
Without noise, *SCR_b_* = 0 dB.

For the GF-3 SAR/GMTI mode, the minimum detectable velocity (MDV) is about 5 m/s and the interferometric phase caused by target movement is 32.4°. On the condition that there is no noise and *SCR_b_* is 0 dB, which means the clutter power equal the moving target power before suppression, SCR after clutter suppressed is shown in Figure 2, where the moving target velocity is 5 m/s. From Figure 2, we can see that if there are no amplitude and phase errors, SCR after clutter suppression is about 50 dB. However, with the increase of amplitude and phase error, SCR decreases rapidly.

**Figure 3:**
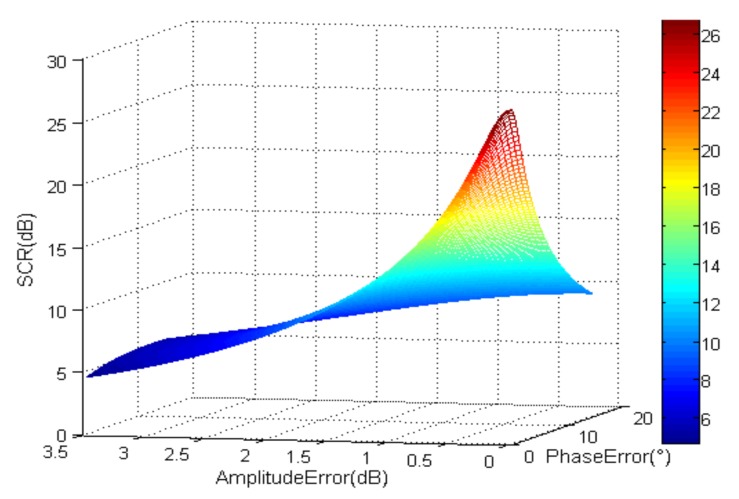
*SNR_b_* = 33 dB, *SCR_b_* = 0 dB.

After clutter suppression, noise is an important influencing factor. Figure 3 shows SCNR after clutter suppression where *SCR_b_* = 0 dB and *SNR_b_* = 33 dB. *SCNR* is only about 27 dB even without amplitude and phase errors.

**Figure 4:**
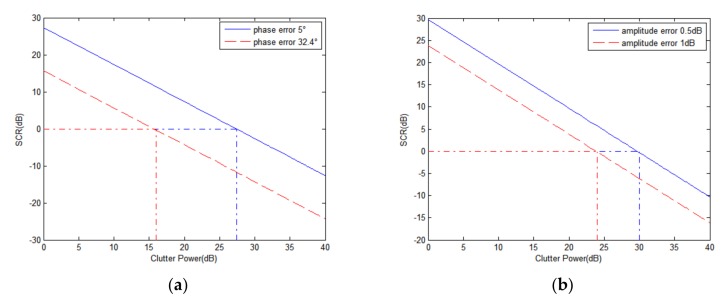
SCR curves with moving target power 10 dB and radial velocity 5 m/s; (**a**) SCR with phase error and without amplitude error; (**b**) SCR with amplitude error and without phase error.

Besides channel error, clutter power is also an important factor. The strong clutter will be not cancelled even with very small amplitude and phase errors between channels. The following examples are used to analyze the effects of clutter power. The curves of SCR after suppression changing with clutter power are shown in Figure 4, where the moving target power is 10 dB and the radial velocity is 5 m/s. To better understand, we added the red dotted lines in Figure 4, which are used to find the corresponding clutter power when SCR = 0 dB, which means that the suppressed clutter is as strong as the moving target.

In Figure 4a, the SCR curves at 5° and 32.4° of phase errors are drawn respectively. Under the condition that the phase error is 5°, the SCR drops to 0 dB when the clutter power is 27.4 dB. This means that when the clutter power exceeds 27.4 dB, the suppressed clutter is stronger than the moving target. If the phase error increases to 32.4°, the SCR drops to 0 dB even when the clutter power is 16 dB. In Figure 4b, the SCR curves at 0.5 dB and 1 dB amplitude error are drawn, respectively. On the condition of 0.5 dB amplitude error, the SCR drops to 0 dB when the clutter power is 30 dB. If the amplitude error increases to 1 dB, the SCR drops to 0 dB when the clutter power is 24 dB. The results are summarized in Table 1.

From Table 1 and Figure 4, it can be seen that the strong clutter component has great impact on the moving target detection. Therefore, the strong clutter component requires particular attention in the process of clutter suppression.

**Figure 5:**
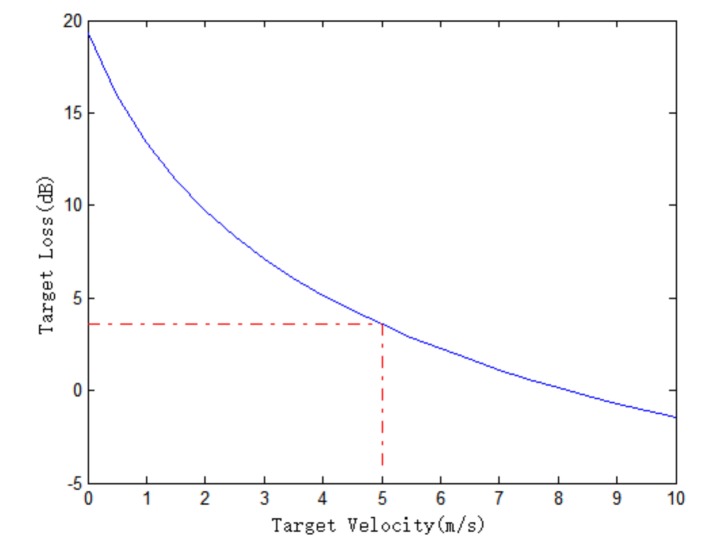
Target loss with amplitude error 0.5 dB and phase error 5° between channels.

Moving targets, especially slow-moving targets, will be partially suppressed when the clutter suppressing operation is carried out. The lower the target velocity, the greater the target loss. This is because the low target velocity leads to a small interferometric phase between channels, and the target is more suppressed. This can be validated by Equations (3) and (4). The curve of the target loss changing with target radial velocity is shown in Figure 5, where the amplitude error is 0.5 dB and the phase error is 5° between channels. It can be seen that the target loss decreases as the target radial velocity increases. The red dotted lines in Figure 5 are used to find the corresponding target loss when the target radial velocity is 5 m/s. The target loss is 3.8 dB when the radial velocity is 5 m/s.

## 3. Algorithm Description

**Figure 6:**
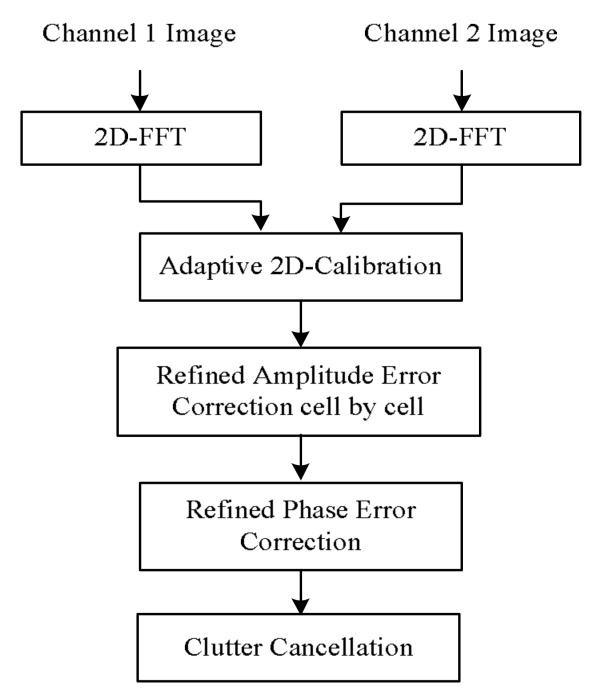
Algorithm flow chart.

For the dual-channel radar, the channel error correction is one of the key issues when the clutter cancellation operation is carried out. The existence of the error will lead to a decline in clutter suppression ability and affect the moving target detection results. The algorithm proposed in this paper is devoted to solving this problem. The proposed algorithm includes three technique steps. The first step is the adaptive 2D-channel calibration. The second step is the refined amplitude error correction cell by cell. The third step is the refined phase error correction, which mainly corrects strong clutter phase error. When channel error is all corrected, almost all of the ground clutter is suppressed greatly and moving targets are detected. The algorithm flow chart is shown in Figure 6. In the following sections, the algorithm is described in detail.

### 3.1. Adaptive 2D-Channel Calibration

Channel balance should be performed before clutter suppression. One adaptive two-dimensional calibration method has been proposed by Ender in [21]. Here, we propose an updated adaptive 2D-calibration method. First, the two channels images are transformed into the 2D-spectrum by FFT. Then, one channel’s data is used as a reference to calibrate the other channel’s data. The model between the reference channel data and pre-calibration channel data is expressed by:

(5) $$Z_{1}(f_{r},f_{d})=Z_{2}(f_{r},f_{d})H(f_{r},f_{d})$$

where $Z_{1}(f_{r},f_{d})$ and $Z_{2}(f_{r},f_{d})$ denote respectively the reference channel data and the pre-calibration channel data, $H(f_{r},f_{d})$ denotes range error and Doppler error model between channels, *f_r_* denotes range frequency, and *f_d_* denotes Doppler frequency.

From Equation (5), according to the least squares criterion (LS), we get:

(6) $$\iint {\left|Z_{1}(f_{r},f_{d})-Z_{2}(f_{r},f_{d})H(f_{r},f_{d})\right|}^{2}df_{r}df_{d}$$

Discretize Equation (6), and we get:

(7) $$\min\sum_{n}\sum_{m}{\left|Z_{1}(mdf_{r},ndf_{d})-Z_{2}(mdf_{r},ndf_{d})H(mdf_{r},ndf_{d})\right|}^{2}$$

where *m* = 1, 2, …, *M*, *M* is the number of range cell of , *n* = 1, 2, …, *N*, *N* is the number of Doppler cell. In the practical application, computer iteration is used instead of solving Equation (7) to complete the calibration of the pre-calibration channel data.

When *Z*_2_ is calibrated, the two channels are almost matched. What needs to be emphasized is that the method calibrates both amplitude and phase difference at the same time. It strengthens the stability and reliability of clutter cancellation.

### 3.2. Refined Amplitude Correction

During the processing of the adaptive 2D-calibration, the amplitude and phase difference of most of the clutter component in the two channels is calibrated. However, due to the existence of random errors and noise, there are still some small amplitude and phase differences between the channels. Therefore, some strong clutter, for example, buildings and bridges, cannot be cancelled completely during clutter suppression and are counted as moving targets during detection. Considering the problem, channels error has to be corrected further. In this section, based on [22], the amplitude error between channels is analyzed and corrected cell-by-cell in the image domain.

First, the two channels data are transformed into the image domain by 2D-IFFT. Then, we calculate the same cell amplitude value of both the reference channel and the pre-calibration channel, which has been calibrated by the adaptive 2D-calibration, respectively. Then the cell amplitude value of the reference channel is divided by the same cell amplitude value of the pre-calibration channel as the following equation:

(8) $$\tilde{A}(m,n)=\frac{\left|Z_{1}(m,n)\right|}{\left|{\tilde{Z}}_{2}(m,n)\right|}$$

where $\tilde{A}(m,n)$ denotes the amplitude ratio of the two channels, $Z_{1}(m,n)$ denotes the reference channel data, ${\tilde{Z}}_{2}(m,n)$ denotes the calibrated channel data by the adaptive 2D-calibration, *m* is range cell and *n* is the azimuth cell.

In the following, multiply the corresponding cell data of the calibrated channel by the amplitude ratio as in the following equation:

(9) $${\tilde{\tilde{Z}}}_{2}(m,n)={\tilde{Z}}_{2}(m,n)\cdot \tilde{A}(m,n)$$

where ${\tilde{\tilde{Z}}}_{2}(m,n)$ denotes the calibrated channel data by the adaptive 2D-calibration and the refined amplitude error correction.

Traverse all the cells using the above operations, and the amplitude error between channels is corrected.

### 3.3. Refined Phase Correction

Although the amplitude error between two channels has been corrected completely, a small phase error still exists. Even a very small phase error between channels will result in the strong clutter left. To get satisfying results, a refined phase correction method is proposed in this section.

In the echo signal, the noise component causes de-correlation of the image between channels. The high clutter–noise ratio (CNR) increases the image correlation. Therefore, strong scatters are selected to complete refined phase correction. The selected strong scatters should meet the two conditions: the amplitude is higher than threshold 1 and the phase is lower than threshold 2. Threshold 1 is determined to make sure that the amplitude of the selected strong scatter is in the top 3% to 6% of the whole data. The percentage depends on how many strong scattering points are in the image.

Threshold 2 is determined by the minimum detectable velocity of the SAR/GMTI sensors and is obtained by the following equation:

(10) $$\varphi_{\mathrm{th}}=\frac{2\pi }{\lambda }\cdot v_{MDV}\cdot \frac{d}{V_{S}}$$

where $\varphi_{\mathrm{th}}$ denotes threshold 2, also called the phase threshold, *v_MDV_* is the minimum detectable velocity, *d* is the aperture displacement, *V_S_* is the satellite velocity, and *λ* is wavelength. Calculate the phase error by the following equation:

(11) $$\Delta \tilde{\varphi }(m,n)=angle(Z_{1s}(m,n))-angle({\tilde{\tilde{Z}}}_{2s}(m,n))$$

where angle(∙) means returning phase angles, $Z_{1s}(m,n)$ denotes the selected clutter from the reference channel, and ${\tilde{\tilde{Z}}}_{2s}(m,n)$ denotes the selected clutter from the calibrated channel by the first two steps. Using $\Delta \tilde{\varphi }(m,n)$ to multiply ${\tilde{\tilde{Z}}}_{2}(m,n)$ as the following equation:

(12) $${\tilde{\tilde{\tilde{Z}}}}_{2}(m,n)={\tilde{\tilde{Z}}}_{2}(m,n)\cdot \exp(j\Delta \varphi (m,n))$$

where ${\tilde{\tilde{\tilde{Z}}}}_{2}(m,n)$ denotes the calibrated channel data by the adaptive 2D-calibration, the refined amplitude error correction and the refined phase correction. Traverse all the cells using the above procedure, and the phase error between channels can be corrected accurately.

Until now, the amplitude and phase errors between channels have been corrected gradually. Clutter is cancelled by subtracting the corrected channel data from the reference channel data and moving targets can be separated from ground clutter.

## 4. Results and Discussion

The dual-channel real spaceborne SAR/GMTI data acquired from GF-3 SAR satellite are used to validate the proposed algorithm. The system parameters are shown in Table 2. Two sets of experimental results are given below.

### 4.1. Experiment 1

**Figure 7:**
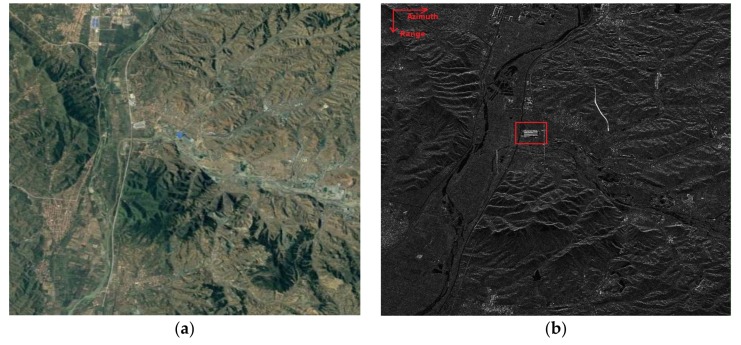
The intercepted scene from GF-3 SAR; (**a**) the aerial view; (**b**) the SAR image.

We intercepted a scene with 2801 range cells and 2501 azimuth cells along the Datong–Qinhuangdao railway line between Shanxi province and Hebei province in China. Figure 7a shows an aerial view of the intercepted scene, where there are a railway and highways. Figure 7b provides the SAR image of the intercepted scene, where two running trains are recognized in the scene. The dynamic range of the whole image is very large. Some pixels are strong scatters, for example, man-made structures, and some pixels are close to noise level, for example, rivers and lakes. Therefore, it is difficult to suppress clutter effectively by conventional methods.

**Figure 8:**
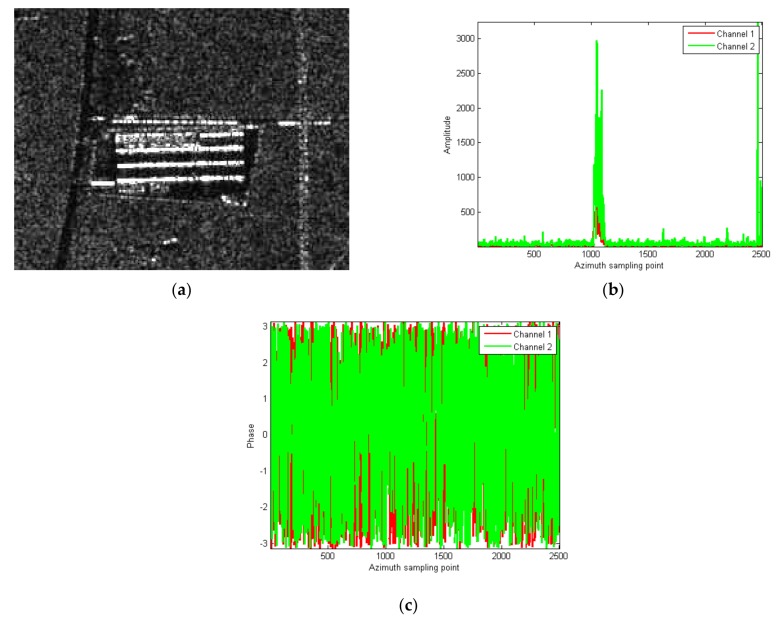
(**a**) The partly intercepted SAR image including artificial construction; (**b**) the amplitude slice including the strong scattering region along azimuth; (**c**) the phase slice including the strong scattering region.

In order to verify the validity of this proposed algorithm, the strong artificial construction area marked in the red box of Figure 7b is extracted, and shown in Figure 8a. One amplitude and phase slice of Figure 7, Figure 7b including the strong scattering region along azimuth are extracted, as shown in Figure 8b,c. As can be seen, the strong scattering points are mainly concentrated at the sampling cells of 1020–1120.

**Figure 9:**
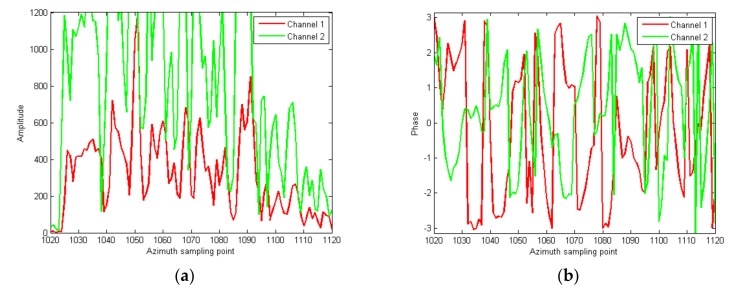
Local amplified amplitude and phase slices of the strong scattering region; (**a**) the amplitude slice; (**b**) the phase slice.

In order to observe in detail, the amplitude and phase of the strong scattering point region in cells of 1020–1120 are amplified and displayed, as shown in Figure 9. It can be seen that there are great differences of both amplitude and phase between channels.

**Figure 10:**
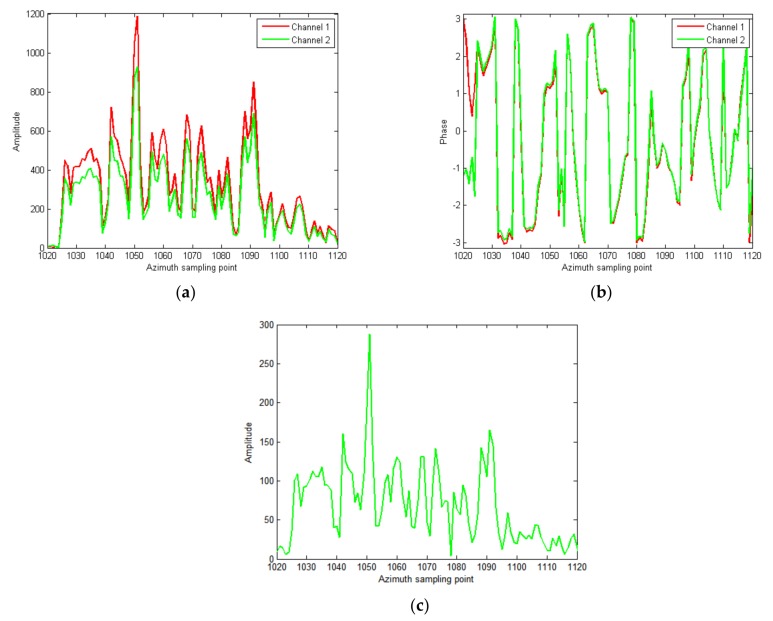
Slices of the strong scattering region after the adaptive 2D-channel calibration; (**a**) the amplitude slice; (**b**) the phase slice; (**c**) the cancelation result.

In the following, the proposed algorithm is used to suppress clutter. First, the 2-D adaptive channel calibration is performed to calibrate the channel difference. The results are shown in Figure 10. It can be seen from Figure 10a,b that the phase consistency between the two channels is good, but the amplitude difference between the two channels is still large. In Figure 10c, after clutter cancellation, the remaining clutter is still strong, and is suppressed by about 12 dB.

**Figure 11:**
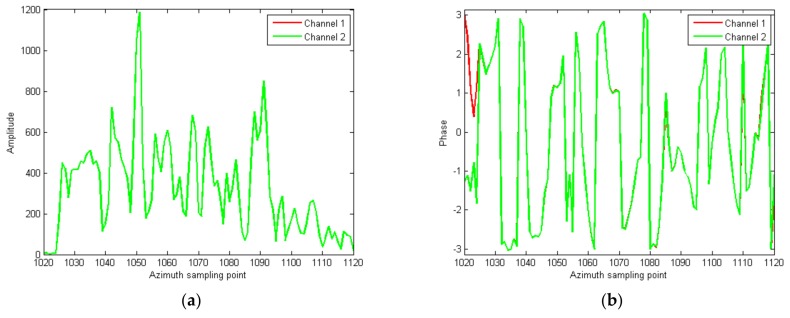

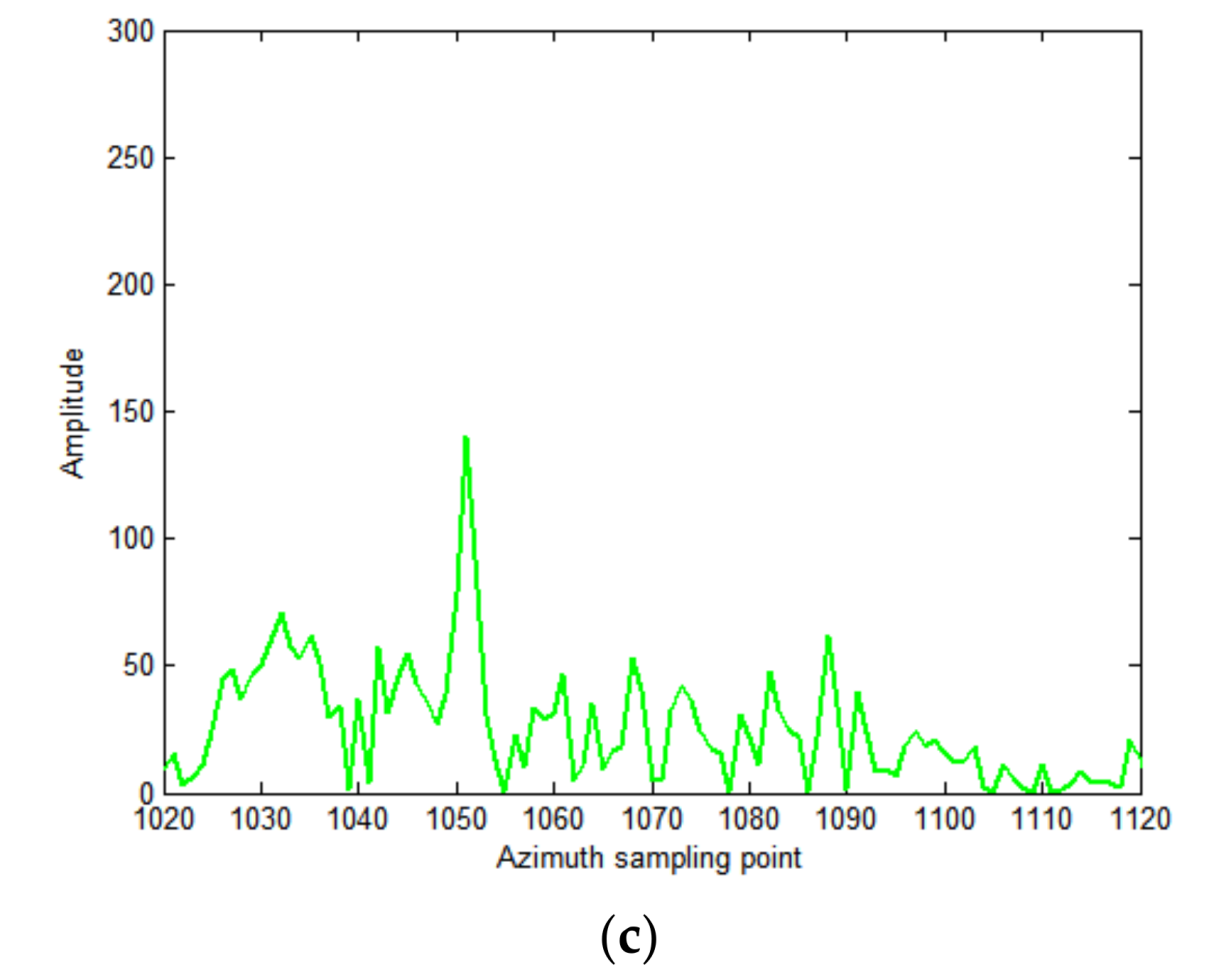
Slices of the strong scattering region after refined amplitude correction cell by cell; (**a**) the amplitude slice; (**b**) the phase slice; (**c**) the cancellation result.

Then the refined amplitude correction is performed cell by cell. The results are shown in Figure 11. The amplitude difference between channels is completely removed, as shown in Figure 11a. The phase difference between channels is very small, except that of the cells 1020–1024, as shown in Figure 11b. In Figure 11c, on the basis of the previous step, the clutter is once more suppressed by about 6 dB. Some clutter is still strong, and is possible to detect a moving target.

**Figure 12:**
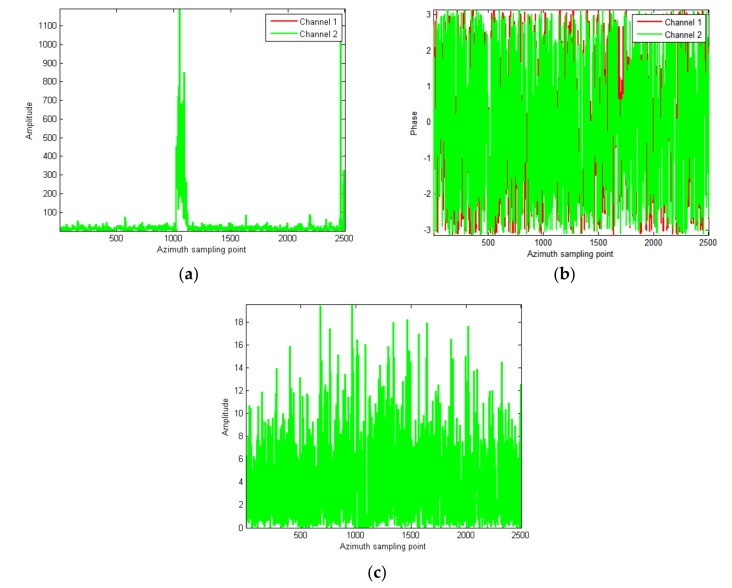
The large image slices after the refined phase correction; (**a**) the amplitude slice; (**b**) the phase slice; (**c**) the cancellation result.

At last, the refined phase correction is performed where the phase threshold is set according to GF-3 MDV (5 m/s). In order to better reflect the clutter suppression effect, the large image slice including strong scattering region is shown in Figure 12. The amplitude slice is shown in Figure 12a, where there is no difference between channels. The phase slice is shown in Figure 12b, where there is some difference between channels, caused mainly by noise. In some regions, the echo energy is weak, and the phase consistency is poor because of the influence of noise. The clutter cancellation result is shown in Figure 12c. It can be seen that the clutter is suppressed to the level of noise and the maximum clutter suppression ratio reaches 37.5 dB.

**Figure 13:**
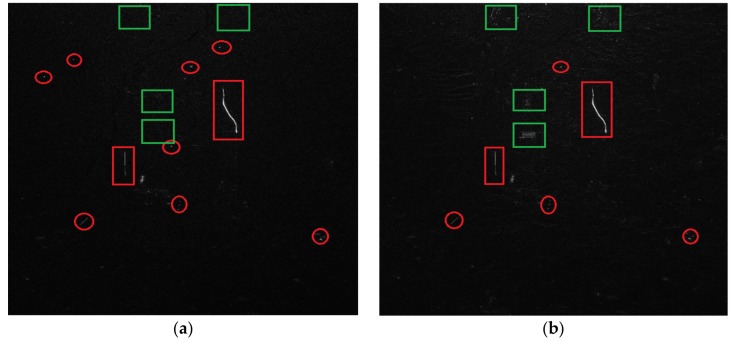
Clutter suppression results of the whole image; (**a**) using the proposed algorithm; (**b**) using the DPCA technique.

The clutter suppression result of the large image using the proposed algorithm is shown in Figure 13a. The conventional DPCA technique was also performed, and the result is shown in Figure 13b. In Figure 13a, almost all the clutter, including strong clutter, was cancelled except the running trains (marked with red rectangles), some suspected moving targets (marked with red circles) and some noise. In Figure 13b, more strong clutter, which is marked with green rectangles, still remains besides the trains and some suspected moving targets. However, there is no clutter in the same regions of Figure 13a. From Figure 13a,b, we can see that the proposed algorithm has a better performance in clutter suppression.

There are two reasons for the poor performance of clutter suppression in Figure 13b. The first is that the experiment data does not adhere to the DPCA condition. In the actual system, due to the changing satellite velocity and the restricted PRF, the DPCA condition is hard to satisfy, resulting in more clutter remaining. The second is caused by the amplitude and phase error between channels. In fact, even if the DPCA condition is satisfied at a moment in the satellite operation, different channel transfer functions will result in the amplitude and phase error between channels, which further aggravates the result for clutter suppression. With the proposed algorithm in the paper, we can get a better result for clutter suppression without adherence to the DPCA condition.

**Figure 14:**
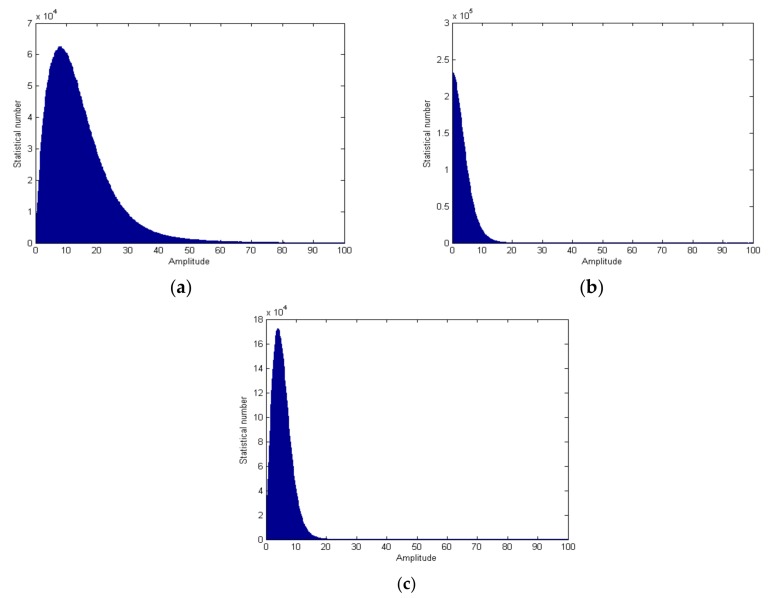
Histogram of data (amplitude in 0–100) before and after clutter suppression; (**a**) before clutter suppression; (**b**) after clutter suppression with the proposed algorithm; (**c**) after clutter suppression with the DPCA technique.

In order to see the clutter suppression effect more clearly, we analyze the data histogram distribution with amplitude in 0–100 (on behalf of the scope of the vast majority of clutter in the scene) before and after clutter suppression, as shown in Figure 14a,b. It can be seen that the clutter is effectively suppressed. By comparing the average power before and after clutter suppression, we find that the clutter is suppressed by 12.83 dB on average. For weak scattering points, such as rivers, its echo intensity is close to noise level, so the clutter suppression ratio is very small, and close to 0 dB. For strong scattering points, such as artificial buildings, the clutter suppression ratio can reach over 35 dB.

We also analyze the data histogram distribution after clutter suppression with the DPCA technique, as shown in Figure 14c. We can see from Figure 14c, that more clutter is left. The clutter is suppressed by 9.15 dB on average. By comparing Figure 1 and Figure 14b,c, the clutter suppression performance of the proposed algorithm is better than that of the DPCA technique.

### 4.2. Experiment 2

**Figure 15:**
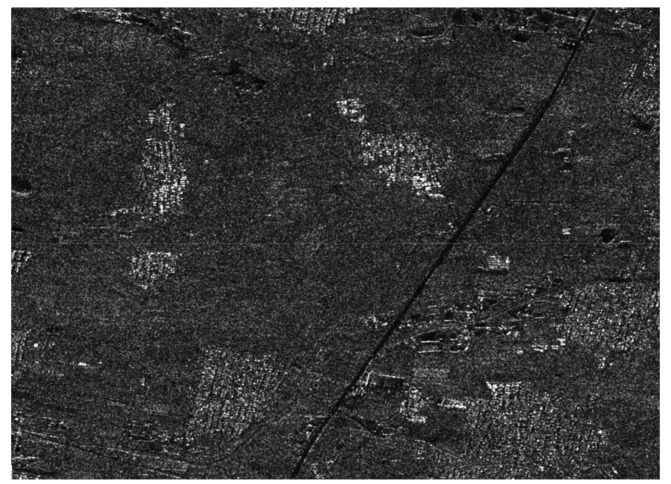
The intercepted GF-3 SAR image.

In order to further verify the proposed algorithm in this paper, the other scene of GF-3 was intercepted, as shown in Figure 15. In the image, besides man-made structures and crops, there is a road in the right part.

**Figure 16:**
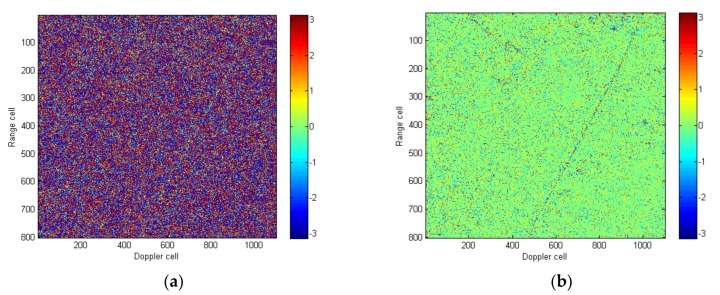
(**a**) The interferometric phase without correcting error; (**b**) the interferometric phase with error corrected using the algorithm proposed in the paper.

First, the interferometric phase of the two channels was drawn, which shown in Figure 16a. As can be seen from the Figure, the interferometric phase is evenly distributed in (−π, π), and if the clutter suppression is carried out directly, there will be a large amount of clutter to be left. After the proposed algorithm was performed on the image, the interferometric phase is shown in Figure 16b. As can be seen from the Figure, the interferometric phase concentrates around 0°, which shows high consistency between channels.

**Figure 17:**
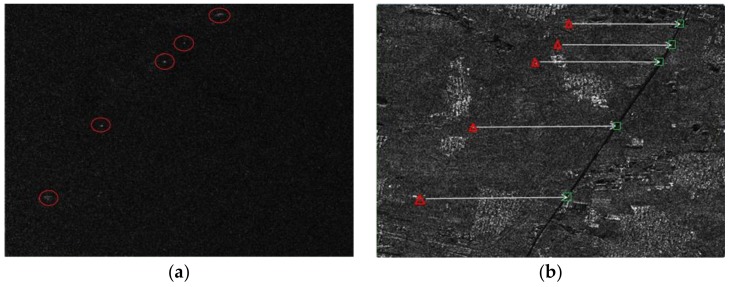
(**a**) The result after clutter suppressed; (**b**) relocation map of the moving targets.

After the channel error is corrected, the clutter suppression result is shown in Figure 17a. As can be seen from the Figure, clutter is almost cancelled. Besides noise, five suspected moving targets remain, marked in red circles. Using the CFAR technique, the five targets are detected. In Figure 17b, these moving targets location are marked with red triangles. According to prior knowledge, the moving targets are thought to be moving on the road, so their relocation positions are marked on the road with green rectangles.

## 5. Conclusions

The GF-3 SAR/GMTI experiment is a dual-receiving channel working mode. In this paper, the clutter suppression principle and model are described and the influence of amplitude and phase error on clutter suppression is analyzed. A novel algorithm of clutter suppression based on high-accuracy amplitude and phase error correction is proposed, which is suitable for the GF-3 SAR sensor. Firstly, the adaptive two-dimensional (2D) channel calibration is performed to calibrate the difference between channels. Then, the refined amplitude correction is performed cell-by-cell to balance the two channels amplitude. At last, the refined phase correction based on the selected strong clutter is performed to correct the phase error. Through these procedures, the channel error is corrected gradually. Compared with conventional techniques, the proposed algorithm has better performance in suppressing strong clutter, especially in artificial buildings, bridges, etc. The real data processing results of the GF-3 SAR system show that the proposed algorithm can obtain satisfactory results with strong clutter suppression.

## Figures and Tables

**Table 1:** Examples for illustrating the effects of clutter power.

Condition	Clutter Power	SCR after Clutter Suppressed
*A* = 0 dB, *θ* = 5°	27.4 dB	0 dB
*A* = 0 dB, *θ* = 32.4°	16 dB	0 dB
*A* = 0.5 dB, *θ* = 0°	30 dB	0 dB
*A* = 1 dB, *θ =* 0°	24 dB	0 dB

**Table 2:** GF-3 SAR system parameters.

Parameters	Value	Parameters	Value
Centre frequency	5.4 GHz	Sensor velocity	7480 m/s
Beam center look angle	32.2°	Signal bandwidth	60 MHz
Along-track baseline	3.75 m	Pule repetition frequency	2372 Hz
